# Smoking-related bladder cancer burden from 1990 to 2021: An age-period-cohort analysis of the global burden of disease study

**DOI:** 10.18332/tid/204744

**Published:** 2025-06-12

**Authors:** Qingqing Yu, Bing Li, Hao Lin, Chao Sun, Xinyue Yang, Zhiqiang Zhang

**Affiliations:** 1 PKUCare Luzhong Hospital, Zibo, China; 2 Department of Urology, Tianjin Medical University General Hospital, Tianjin, China; 3 Department of Orthopedics, Tianjin Medical University General Hospital, Tianjin, China; 4 Graduate School of Tianjin Medical University, Tianjin Medical University, Tianjin, China

**Keywords:** global burden of disease, bladder cancer, smoking, ageperiod-cohort analysis

## Abstract

**INTRODUCTION:**

Bladder cancer is common in the elderly, with smoking as a major risk factor. This study assesses the global burden of bladder cancer attributable to smoking, using data from 204 countries and regions (1990–2021).

**METHODS:**

Mortality, age-standardized mortality rates (ASMR), disability-adjusted life years (DALYs), and age-standardized DALY rates (ASDR) were extracted from the Global Burden of Disease (GBD) 2021 study. The burden of smoking-related bladder cancer was analyzed by age, gender, and sociodemographic index (SDI).

**RESULTS:**

Between 1990 and 2021, global deaths due to smoking-related bladder cancer increased by 42.9%, while disability rose by 31.0%. In 2021, the global number of bladder cancer deaths due to smoking reached 58766 (95% UI: 49381–70891). Despite these increases, both ASMR and ASDR declined globally. Males experienced a higher increase in mortality and disability, with rates approximately 10 times higher than females. Middle SDI countries saw the largest rise in burden. The annual decline in mortality was 2.06%, greater in males than females.

**CONCLUSIONS:**

The burden of smoking-related bladder cancer is higher in males, middle-aged and elderly individuals, and medium to high SDI countries. Targeted prevention and health policies are crucial to reducing the disease’s impact on populations and healthcare systems.

## INTRODUCTION

As the global population continues to grow and the aging population expands, the incidence of cancer is rising annually. Cancer has become a major public health issue worldwide. Cancer is reported to be the second leading cause of death in the United States. In 2023, the US recorded 1958310 new cancer cases and 609820 cancer-related deaths^1^. Bladder cancer is a common and high-incidence urological malignancy^2^ that significantly impacts human health and places a heavy burden on healthcare systems. In 2019, the Global Burden of Disease (GBD) study reported 524300 new bladder cancer cases globally, leading to 166440 deaths^3^. According to the American Cancer Society, bladder cancer (BC) ranked as the fourth most common cancer and the eighth leading cause of cancer-related deaths among men in the US in 2022^1^. Further primary prevention efforts are essential to alleviate the medical and economic burden of bladder cancer on populations and healthcare systems.

Contemporary overviews of BC epidemiology and current evidence on BC risk factors indicate that bladder cancer is a multifactorial disease, with both genetic and environmental factors contributing to its development. Smoking and certain occupational exposures are the most established risk factors^4^. Inhalation of tobacco smoke is the most common risk factor for bladder cancer, accounting for approximately 50% of cases^5^. Tobacco smoke contains 70 known carcinogens. Although the primary chemicals responsible for bladder cancer remain uncertain, studies suggest that aromatic amines such as 4-aminobiphenyl (4-ABP) and 2-naphthylamine (2-NA) in tobacco are linked to the disease’s development in smokers^6^. Although evaluations have been made based on the 2019 GBD data, there is a lack of studies based on the 2021 GBD data^7-9^. We analyzed the latest GBD data with the goal of developing more effective policies to better address the challenges posed by smoking-related bladder cancer.

## METHODS

### Data sources and study design

This study uses data from the GBD 2021, following the project’s methodological framework and analysis strategy. GBD 2021 provides annual epidemiological estimates for 371 diseases and injuries worldwide from 1990 to 2021.Specifically, the study focuses on the impact of smoking on bladder cancer, analyzing related indicators such as mortality, disability-adjusted life years (DALYs), age-standardized mortality rates (ASMR), and age-standardized DALY rates (ASDR). Data were collected from the Global Health Data Exchange (GHDx) query tool, which provides detailed information categorized by year, age group, region, and country. The International Classification of Diseases, 10th edition (ICD-10) codes for bladder cancer-related deaths and non-fatal diseases in GBD 2021 include C67-C67.9, D09.0, D30.3, D41.4-D41.8, and D49.4. GBD employs a comparative risk assessment approach based on a causal framework and a hierarchical structure of risk factors. These factors are categorized into four levels (from the broadest, level 1, to the most specific, level 4) to estimate their relative contribution to disease burden.

### Definition of tobacco exposure

In this study, smoking is defined as a level 3 risk factor according to the GBD definition, which includes the smoking rates of current and former smokers. Smoking is defined as the current or past occasional or daily use of tobacco products, including pipes, cigarettes, cigars, waterpipes, bidis, and other locally used tobacco products. For current smokers, daily cigarette consumption and cumulative years of smoking exposure are used as metrics. For former smokers, the distribution of years since quitting is estimated.

### Sociodemographic index classification

The study also incorporates the sociodemographic index (SDI) for each country and region. SDI is a composite measure based on lag-distributed income (LDI), average education level (EDU15+), and total fertility rate (TFU25). SDI values range from 0 to 1, with higher values indicating higher socio-economic development levels in a region. In the analysis, all countries and regions are grouped into five categories based on their 2021 SDI values, ranging from high to low socio-economic levels.

### Outcome measures and metrics

DALYs, a key indicator of disease burden, combine the health losses due to both death and disability. Specifically, DALYs consist of two components: Years of life lost (YLLs) and years lived with disability (YLDs). YLLs measure the years of life lost due to death by calculating the difference between the number of deaths and the expected life expectancy for the group. YLDs are calculated by multiplying the number of people with a disease by the disability weight (which indicates the severity of the disease) and the duration of the disease, to estimate years of disability caused by illness or injury. The total DALYs are the sum of YLLs and YLDs, providing a comprehensive reflection of the impact of a disease or health problem on the population’s health. ASMR and ASDR are age-standardized, making the data comparable across different regions or time periods, thus eliminating biases due to differences in age structures. The calculation of ASMR and ASDR helps accurately reflect mortality risks and health losses across different populations.

### Statistical methods

This study uses the age-period-cohort (APC) model, widely applied in epidemiology, to analyze mortality trends across different birth cohorts, time periods, and age groups. The APC model is commonly used in chronic disease research and helps describe long-term trends in disease mortality over time, revealing the impact of age-related biological, social factors, and technological advances on disease burden. By incorporating Lexis diagrams, the APC model estimates the cumulative effects resulting from changes in age, time period, and birth cohort. The APC model’s input data is sourced from GBD 2021, covering mortality and DALYs data for smokers from 1990 to 2021, organized into annual data by five-year intervals. The data are aggregated by country and region, categorized by age groups, based on mortality and DALYs. To ensure data representativeness and stability, five-year age groups (e.g. 50–54 to 90–94 years) were used, as mortality numbers for those aged <50 years and >95 years are low. Birth years are divided into five-year intervals (e.g. 1890–1899 to 1960–1969) to allow for a more detailed observation of trends over different periods. The fitted APC model calculates the annual changes in mortality rates resulting from the combined effects of age, period, and birth cohort, expressing this as a net drift with annual percentage changes. Net drift is computed by combining calendar time effects and birth cohort effects, reflecting the dynamic changes in disease burden over time. In this study, for estimates directly extracted from the GBD study (such as incidence rates, mortality rates, etc.), we report 95% uncertainty intervals (95% UI); for results obtained through our own statistical modeling (such as estimated annual percentage change [EAPC]), we report 95% confidence intervals (95% CI). All statistical analyses were conducted using R software (version 4.3.1).

## RESULTS

### Global trends

From 1990 to 2021, the global number of bladder cancer deaths due to smoking increased by 42.9% (95% UI: 25.7–68.3), and the total disability burden (DALYs) increased by 31.0% (95% UI: 14.5–56.8). From 1990 to 2021, the global number of bladder cancer deaths due to smoking increased by 42.9% (95% UI: 25.7–68.3), and the total disability burden (DALYs) increased by 31.0% (95% UI: 14.5–56.8). In 2021, the global number of bladder cancer deaths due to smoking reached 58766 (95% UI: 49381–70891) and the total number of disabilities (DALYs) reached 1238303 (95% UI: 1044303–1478221) (Tables 1 and 2).

**Table 1:** Mortality among people with bladder cancer attributable to smoking by sex and SDI from 1990 to 2021

	*Deaths*		*All-age mortality*		*Age-standardized mortality*		*Net drift of mortality* *% per year (95% CI)*
	*Number in 2021* *n (95% UI)*	*Change in number* *1990–2021* *% (95% UI)*	*Rate in 2021* *per 100000* *(95% UI)*	*Percent change* *1990–2021* *% (95% UI)*	*Rate in 2021* *per 100000* *(95% UI)*	*Percent change* *1990–2021* *% (95% UI)*	
**Global**	58766.9 (49381.3–70891.7)	42.94 (25.65–68.33)	0.7 (0.6–0.9)	-3.39 (-15.07–13.77)	0.7 (0.6–0.8)	-38.09 (-45.4 – -27.4)	-2.06 (-2.14 – -1.98)
**Sex**							
Male	53090.1 (44382.3–64164)	45.76 (25.67–75.08)	1.3 (1.1–1.6)	-1.13 (-14.76–18.76)	1.4 (1.2–1.8)	-38.98 (-46.79 – -27.61)	-2.11 (-2.2 – -2.03)
Female	5676.7 (4571.3–6958.5)	21 (10–31.47)	0.1 (0.1–0.2)	-18.51 (-25.93 – -11.47)	0.1 (0.1–0.1)	-48.48 (-52.97 – -44.26)	-2.33 (-2.59 – -2.08)
**SDI**							
High	19185.3 (15484.6–23062.3)	15.99 (7.18–24.94)	1.8 (1.4–2.1)	-6.75 (-13.84–0.44)	0.8 (0.7–1)	-42.53 (-46.49 – -38.74)	-2.05 (-2.22 – -1.89)
High-middle	19649.7 (16415.8–23607.5)	38.36 (20.06–63.65)	1.5 (1.3–1.8)	12.84 (-2.09–33.47)	1 (0.8–1.2)	-34.22 (-43.02 – -21.91)	-1.85 (-1.98 – -1.71)
Middle	13622.6 (10819.7–17705)	101.1 (53.27–185.12)	0.6 (0.4–0.7)	41.5 (7.85–100.62)	0.6 (0.4–0.7)	-28.37 (-45.04–0.47)	-1.72 (-1.88 – -1.57)
Low-middle	5102.6 (4109.2–7284.7)	75.77 (37.43–158.68)	0.3 (0.2–0.4)	6.26 (-16.92–56.38)	0.4 (0.3–0.6)	-28.72 (-44.11–2.24)	-1.87 (-2.11 – -1.63)
Low	1127.4 (911.3–1421.5)	77.05 (42.14–131.73)	0.1 (0.1–0.1)	-20.57 (-36.23–3.96)	0.3 (0.2–0.3)	-22.65 (-38.27–0.99)	-1.07 (-1.58 – -0.56)

The all-age mortality is equivalent to the crude mortality rate. The net drifts are estimates derived from the age-period-cohort model and signify the overall annual percentage change in mortality, encompassing the effects from calendar time and successive birth cohorts.

**Table 2:** DALYs among people with bladder cancer attributable to smoking by sex and SDI from 1990 to 2021

	*Disability*		*All-age DALYs*		*Age-standardized DALYs*		*Net drift of DALYs* *% per year (95% CI)*
	*Number in 2021* *n (95% UI)*	*Change in number* *1990–2021* *% (95% UI)*	*Rate in 2021* *per 100000* *(95% UI)*	*Percent change* *1990–2021* *% (95% UI)*	*Rate in 2021* *per 100000* *(95% UI)*	*Percent change* *1990–2021* *% (95% UI)*	
**Global**	1238303.2 (1044303.3–1478221)	30.97 (14.48–56.75)	15.7 (13.2–18.7)	-11.48 (-22.63–5.95)	14.3 (12.1–17.1)	-40.78 (-48.01 – -29.44)	-1.98 (-2.04 – -1.92)
**Sex**							
Male	1126415.8 (945017.8–1348080.9)	32.98 (14.55–61.82)	28.4 (23.9–34)	-9.8 (-22.3–9.76)	28.5 (23.9–34.2)	-41.29 (-49.09 – -29.23)	-2.03 (-2.1 – -1.97)
Female	111887.4 (92227.3–132824.1)	13.65 (4.84–22.15)	2.8 (2.3–3.4)	-23.46 (-29.4 – -17.74)	2.4 (2–2.9)	-48.89 (-52.69 – -45.19)	-2.25 (-2.31 – -2.19)
**SDI**							
High	375065.9 (313625.7–439687.7)	4.99 (-1.78–11.96)	34.3 (28.7–40.2)	-15.59 (-21.04 – -9.99)	17.8 (15–20.8)	-44.41 (-47.66 – -41.08)	-1.96 (-2.05 – -1.86)
High-middle	423433.6 (356413.6–505773.6)	25.56 (8.25–49.84)	32.5 (27.3–38.8)	2.4 (-11.72–22.2)	21.1 (17.7– 25.2)	-37.34 (-45.84 – -24.99)	-1.75 (-1.83 – -1.66)
Middle	295301.6 (233936.6–385221.7)	80.57 (36.06–157.77)	12.1 (9.6–15.7)	27.05 (-4.26–81.38)	11.1 (8.8–14.4)	-31.76 (-48.47 – -3.38)	-1.64 (-1.71 – -1.56)
Low-middle	116525.1 (94038.9–165601.1)	64.12 (28.28–151.12)	6.1 (4.9–8.6)	-0.78 (-22.45–51.82)	8.3 (6.7–11.9)	-30.66 (-45.76–3.02)	-1.86 (-1.98 – -1.74)
Low	26247.5 (21113.2–32827.3)	73.97 (38.92–126.77)	2.3 (1.9–2.9)	-21.95 (-37.68–1.74)	5.5 (4.5–6.9)	-22.95 (-38–0.03)	-1.07 (-1.18 – -0.96)

The all-age DALYs is equivalent to the crude disability rate. The net drifts are estimates derived from the age–period–cohort model and signify the overall annual percentage change in DALYs, encompassing the effects from calendar time and successive birth cohorts.

### Age and gender trends

**Figure 1:**
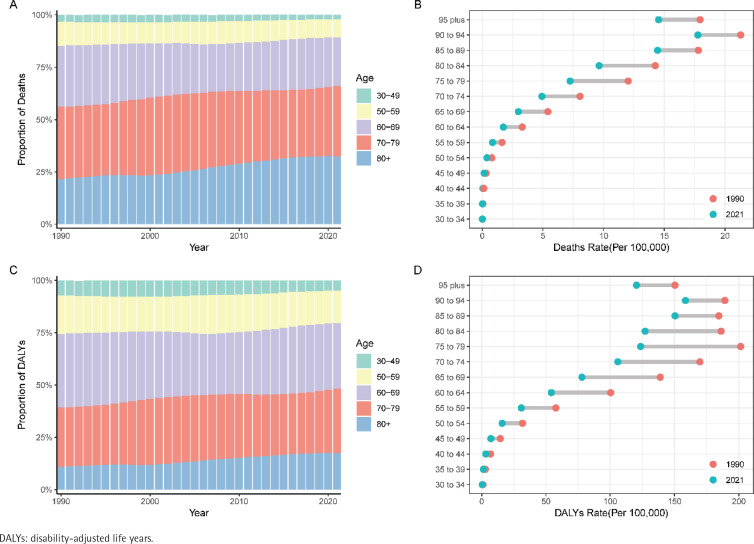
The temporal change of the mortality rate and DALYs in bladder cancer attributable to smoking across age groups: A, C) The relative proportion of bladder cancer mortality and DALYs; B, D) The temporal changes in the mortality rate and DALYs of bladder cancer

Age group analysis shows that bladder cancer mortality is increasingly concentrated in individuals aged ≥80 years (Figure 1A), while DALYs due to smoking shift towards those aged ≥60 years (Figure 1C). Overall, smoking has a significant impact on bladder cancer mortality and DALYs in the elderly population. Notably, from 1990 to 2021, the age group of 75–79 years experienced the largest decline in bladder cancer mortality (1990: 12.0%; 95% UI: 10.3–13.8; 2021: 7.2%; 95% UI: 6.0–8.6) (Figure 1B). Similarly, the age group of 75–79 years showed the largest decrease in bladder cancer DALYs (Figure 1D). Over the past 30 years, the increase in bladder cancer deaths and DALYs due to smoking was much higher in men compared to women, with the growth rate approximately 2.5 times greater in men. By 2021, the absolute number of bladder cancer deaths and DALYs in men was approximately 10 times that of women. Although both age-standardized mortality and DALYs for men and women declined from 1990 to 2021, significant gender differences remain in 2021 (Tables 1 and 2). Additionally, compared to women, smoking men tend to experience bladder cancer death and disability at younger ages, whereas women are more prone to these outcomes at older ages (Supplementary file Figures 1 and 2).

### Trends by sociodemographic index (SDI)

From 1990 to 2021, bladder cancer deaths and DALYs due to smoking followed a growth pattern consistent with global trends across countries of all SDI levels. Among these, countries with a middle SDI showed the most significant increase, with death rates rising by 101.1% (95% UI: 53.3–185.1) and DALYs increasing by 80.6% (95% UI: 36.1–157.8). Meanwhile, the age-standardized mortality rate (ASMR) declined across all SDI levels, with the most significant decrease in high SDI countries. By 2021, countries with middle-high SDI had the highest number of deaths and age-standardized mortality rates, reaching 19649.7 (95% UI: 16415.8–23607.5) and 1.0 per 100000 people (95% UI: 0.8–1.2) (Table 1). Similarly, DALYs and age-standardized DALYs showed a downward trend across different SDI categories, with the largest decline in high SDI countries. However, the total DALYs and age-standardized DALYs in 2021 remained highest in middle-high SDI countries, with 423433.6 (95% UI: 356413.6–505773.6) and 21.1 per 100000 people (95% UI: 17.7–25.2) (Table 2). Global trends show that as SDI levels increase, both ASMR and DALYs due to smoking decline. The decline was most significant in Western Europe (Supplementary file Figure 3). In low SDI countries, the EAPC values are mostly positive, indicating a smaller decline or even a slight increase in ASMR, while in high SDI countries, the EAPC values are mostly negative, indicating a sustained decline in ASMR (Supplementary file Figure 3). The EAPC trends for DALYs are consistent with those for mortality (Supplementary file Figure 4).

### Age group analysis by SDI levels

In high SDI regions, bladder cancer mortality is increasingly concentrated in individuals aged ≥80 years, while DALYs are concentrated in the age group of 50–69 years across all SDI regions. The declining trends in smoking-attributable bladder cancer mortality and DALYs have been significant and consistent in individuals aged >60 years old across all SDI regions (Supplementary file Figures 5 and 6).

### Country trends

From 1990 to 2021, more than half of the countries globally saw a decline in bladder cancer mortality due to smoking, with San Marino, the UK, and Italy showing the most significant decreases. At the same time, most countries showed a decline in bladder cancer DALYs due to smoking, with the largest decreases observed in the UK, Italy, and Spain (Supplementary file Figure 7). However, in 2021, Lebanon (ASMR=2.9/ASDR=58.9 cases per 100000), Armenia (ASMR=2.3/ASDR=50.9 cases per 100000), and Greece (ASMR=2.2/ASDR=46.9 cases per 100000) still had the highest global rates for mortality and DALYs. Although these countries had reduced mortality rates compared to 1990, their DALYs were still higher than in 1990 (Supplementary file Table 1).

### Local drift, age, period, and cohort effects

**Figure 2:**
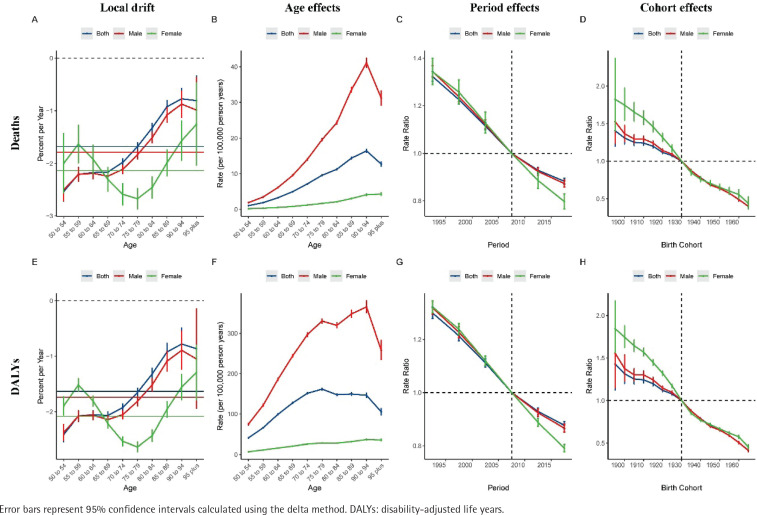
The local drifts, age effects, period effects, and cohort effects of bladder cancer mortality attributable to smoking from 1990 to 2021: A–D) mortality data; E–H) DALYs

Figure 2 provides insights into indicators derived from the age-period-cohort model, including local drift, age effects (represented by longitudinal age curves, describing the natural changes in bladder cancer mortality and DALYs), period effects (showing the relative death risk at different periods), and cohort effects for male and female cohorts (showing relative death risk in the cohort). Overall, according to the age-period-cohort model, the net drift in bladder cancer mortality due to smoking is -2.06% per year (95% CI: -2.41 – -1.98) (Table 1). However, there are differences in the net drift between men and women, with the net drift increasing as age rises in men. In contrast, women aged 55–79 years showed a decreasing trend, while those aged ≥80 years showed an increasing trend. Compared to men (-2.11%; 95% CI: -2.2 – -2.03), the decline in female bladder cancer mortality was slightly lower (-2.33%; 95% CI: -2.59 – -2.08), and a similar pattern was observed for bladder cancer DALYs (men: -2.03% (95% CI: -2.1 – -1.97), women: -2.25%; 95% CI: -2.31 – -2.19). The longitudinal age distribution of bladder cancer mortality by gender shows that mortality gradually increases with age in the smoking population. Although the DALYs results followed a similar growth pattern to male and female mortality, the growth rate in men was significantly higher than in women. Regarding period effects, the patterns of decreasing bladder cancer mortality and DALYs in smoking men and women were similar. Similarly, cohort effects show a positive decline in bladder cancer mortality and DALYs, with a slightly greater decrease in women than in men.

## DISCUSSION

This study, based on the latest GBD 2021 data, analyzes the impact of smoking on the burden of bladder cancer, revealing key trends and risk factors. Our results show that while the global number of bladder cancer deaths attributable to smoking has increased by 42.9% over the past 30 years (95% UI: 25.7–68.3), both the ASMR and the ASDR have declined globally, especially the significant drop in ASDR. This suggests that the burden of bladder cancer has been alleviated in some regions, partly due to a decrease in smoking rates and strengthened public health policies^10^. However, the total death and disability burden from smoking-related bladder cancer varies significantly across different age groups, genders, and countries with varying SDI levels. For example, while bladder cancer mortality in those aged ≥80 years continues to rise, the disability burden (DALYs) from smoking-related bladder cancer has shifted to individuals aged ≥60 years. This indicates that older adults play a crucial role in the smoking-related bladder cancer burden^2,10^. Notably, between 1990 and 2021, the most significant decline in both bladder cancer mortality and disability burden occurred in the age group of 75–79 years, highlighting the positive impact of public health interventions in reducing the burden in older populations.

Compared to previous studies, this research provides a more comprehensive analysis across age, gender, and socioeconomic backgrounds. For instance, Zhang et al.^11^ reported a global increase in bladder cancer burden and noted that despite declining smoking rates, the burden of smoking-related bladder cancer remains high in some regions. This study further emphasizes the larger impact of smoking on men, with the absolute number of bladder cancer deaths and disabilities among men being 10 times higher than in women, and the increase about 2.5 times that of women^12^. This gender disparity is not only reflected in mortality rates but is also particularly prominent in the increase of DALYs^10^. Additionally, this study found that the burden of bladder cancer is more significant in high and middle SDI countries compared to low SDI countries. Despite improvements in health policies and public health measures in these regions, the historically high levels of smoking exposure continue to contribute to the persistent burden of bladder cancer. This finding aligns with the research by Tian et al.^12^ and Huang et al.^13^, suggesting that despite tobacco control policies in high-income countries, the historical accumulation of smoking exposure continues to pose a significant bladder cancer burden.

Numerous studies have reported the link between smoking and bladder cancer risk, including a study from Japan that found an association between smoking and increased bladder cancer risk. After many years of quitting, the risk for former smokers may approach that of never smokers^14^. However, there are few reports on the specific mechanisms underlying this effect. These findings have important implications for public health strategies and clinical practice. First, they emphasize that smoking cessation programs should highlight bladder cancer risk reduction as a significant benefit, particularly for middle-aged smokers who might be more concerned about cancer than cardiovascular disease. Second, the gradual but incomplete risk normalization suggests that former smokers, especially those with longer smoking histories, may benefit from tailored surveillance protocols. Finally, understanding the time-dependent nature of risk reduction after cessation provides valuable information for developing personalized risk assessment tools that could guide screening recommendations based on individual smoking histories. Integrating these insights into comprehensive tobacco control programs could significantly enhance their effectiveness in reducing bladder cancer burden. The primary mechanism is through long-term exposure of bladder epithelial cells to carcinogens in tobacco smoke, causing DNA damage and genetic mutations. Aromatic amines (e.g. 4-ABP, 2-NA) and N-nitroso compounds are excreted in urine, where they come into contact with bladder epithelium and promote the development of bladder cancer^15^. Previous research has identified the N-acetyltransferase (NAT2) gene as being associated with bladder cancer risk. The NAT2 gene encodes an enzyme responsible for metabolizing aromatic amines, which are common carcinogens in tobacco smoke^16^. The largest prospective WBC DNA EWAS study on bladder cancer shows that bladder cancer risk-associated CpG sites are primarily related to smoking behavior. Smoking-related DNA methylation at CpG sites is generally associated with future bladder cancer, with most identified differential methylation regions being directly or indirectly related to smoking^17^.

This study further confirms the relationship between smoking and bladder cancer, finding that the impact of smoking on both mortality and disability burden increases with age, especially in older populations. Long-term smoking exposure not only increases the risk of bladder cancer mortality but also leads to higher disability burden, a trend that is especially pronounced in middle- and high-income countries^13^. Furthermore, the impact of smoking on bladder cancer is not limited to men. While the increase in mortality and disability is greater among men, women in the very old age groups (aged ≥80 years) also show higher mortality and disability rates^10^. This gender difference may stem from historical variations in smoking behaviors between men and women, with women typically initiating smoking habits later than men, as well as potential sex-related physiological factors that influence cancer development. These findings suggest that public health interventions should employ gender-differentiated strategies – this does not mean delaying interventions for women, but rather customizing educational messages, support systems, and health promotion activities to address the specific risk factors and concerns of different gender groups. Smoking cessation interventions for both men and women should be timely and proactive, while intervention designs should thoroughly consider the unique barriers and motivating factors that different genders may face, thereby enhancing intervention effectiveness.

Our study further examined the impact of the SDI on bladder cancer mortality and disability burden. We found a significant decline in ASMR and DALYs in high SDI countries, such as those in Western Europe and North America. This trend can be attributed to the early implementation of anti-smoking policies and the stronger public health systems in these countries. However, in middle- and high-SDI countries, despite a decrease in mortality and disability rates, the overall burden remains high, particularly among the male population. In low SDI countries, while mortality and disability rates have decreased, the overall burden remains heavy. This indicates that low-income countries still face significant challenges in smoking control, early screening, and treatment^4^. This suggests that while smoking control policies have been strengthened in various countries, disparities in economic levels and healthcare resources continue to influence the burden of bladder cancer. Particularly in low- and middle-income countries, the lack of effective smoking cessation programs and early screening methods makes it difficult to reduce the burden of bladder cancer.

### Limitations

While this study is supported by extensive global data from the GBD database, there are still several limitations. First, this study relies on data from public health databases, which may have reporting biases, particularly in low-income countries where incomplete data may lead to an underestimation of the actual burden^18^. Second, although our study accounted for various risk factors, it was not possible to control for all potential confounding factors, particularly individual smoking exposure and genetic susceptibility^5^.Our APC regression analysis includes inherent methodological constraints, particularly the ‘identification problem’ where separating age, period, and cohort effects requires additional mathematical constraints. Additionally, our study addresses only active smoking attribution, excluding secondhand smoke exposure and emerging tobacco products such as e-cigarettes, which may contribute to bladder cancer risk but lack sufficient longitudinal data for analysis.

### Future research

Future research should focus on refining dose-response relationships, examining how smoking intensity and duration interact with genetic factors. Further investigation into specific biological mechanisms of tobacco carcinogens and their metabolic pathways in diverse populations is needed. Critical priorities include evaluating the effectiveness of cessation interventions in reducing bladder cancer risk over time and exploring potential synergistic effects between smoking and other environmental exposures. These targeted research directions offer more meaningful contributions to bladder cancer prevention than studies of already confirmed causal relationships.

## CONCLUSIONS

This study reveals that smoking is a major driver of the bladder cancer burden, particularly among middle-aged and older men and in high SDI countries. Despite a global decline in smoking rates, long-term smoking exposure continues to contribute to the persistent burden of bladder cancer. To effectively reduce the incidence and mortality of bladder cancer, more proactive anti-smoking measures must be implemented, particularly among high-risk groups. Based on our findings, we recommend implementing differentiated tobacco control policies tailored to specific demographic profiles, with age-targeted cessation programs for middle-aged smokers, gender-sensitive intervention approaches, and SDI-stratified strategies that address regional development contexts. Additionally, healthcare systems should integrate bladder cancer risk assessment into routine primary care for current and former smokers while strategically allocating prevention resources toward regions showing stagnant or increasing trends in smoking-attributable bladder cancer burden.

## Supplementary Material



## Data Availability

The data supporting this research are available from the authors on reasonable request.
