# Supplementary material for: Smoking-related bladder cancer burden from 1990 to 2021: An age-period-cohort analysis of the global burden of disease study
Source: Tob Induc Dis. 2025 Jun 12;23:10.18332/tid/204744. doi: 10.18332/tid/204744 (PMC12160010; doi:10.18332/tid/204744)
Supplement: Supplementary file 1 [file TID-23-81-s1.pdf]

Smoking-related Bladder Cancer Burden from 1999 to 2021: An Age–Period–Cohort Analysis of the Global Burden of Disease Study

SUPPLEMENTARY DATA

Table of Contents

1. Supplementary Table 1. Intercountry age-standardized mortality rates and DLAYs for bladder cancer attributable to smoking from 1990 to 2021........2
2. Supplementary figure 1. The temporal change of the mortality rate in bladder cancer attributable to smoking across age groups and sexes........8
3. Supplementary figure 2. The temporal change of the DALYs in bladder cancer attributable to smoking across age groups and sexes........9
4. Supplementary figure 3. The relationship between SDI levels and (A)the age-standardized mortality rate and (B)the estimated annual percentage change of ASMR for bladder cancer attributable to smoking from 1990 to 2021. 10
5. Supplementary figure 4. The relationship between SDI levels and (A)the age-standardized DALYs rate and (B)the estimated annual percentage change of ASDR for bladder cancer attributable to smoking from 1990 to 2021. 11
6. Supplementary figure 5. The temporal change of the mortality rate in bladder cancer attributable to smoking across age groups and SDI........12
7. Supplementary figure 6. The temporal change of the DALYs in bladder cancer attributable to smoking across age groups and SDI........13
8. Supplementary figure 7. Maps showing age-standardized changes in mortality rates (A, B) and DALYs (C, D) among people with bladder cancer attributable to smoking from 1990 to 2021........14

1. **Supplementary Table 1.** Intercountry age-standardized mortality rates and DLAYs for bladder cancer attributable to smoking from 1990 to 2021.

Regions	mortality		DALYs	
	ASMR (per 100,000) in 1990	ASMR (per 100,000) in 2021	ASDR (per 100,000) in 1990	ASDR (per 100,000) in 2021
Afghanistan	0.4 (0.2 to 0.8)	0.5 (0.3 to 0.7)	9.4 (5 to 17.2)	10.5 (5.9 to 15.9)
Albania	0.1 (0.1 to 0.2)	0.1 (0.1 to 0.2)	2.6 (2 to 3.5)	2.3 (1.6 to 3.3)
Algeria	0.7 (0.5 to 1)	0.5 (0.4 to 0.8)	12.7 (8.6 to 16.6)	9.4 (6.6 to 12.9)
American Samoa	0.3 (0.2 to 0.5)	0.4 (0.3 to 0.5)	7.9 (5.8 to 10.8)	9.1 (6.9 to 11.8)
Andorra	2.3 (1.5 to 3.3)	1.1 (0.7 to 1.7)	48.6 (31.8 to 71.4)	24 (14.9 to 35.4)
Angola	0.4 (0.3 to 0.6)	0.4 (0.3 to 0.5)	9.6 (6.5 to 14.1)	8.2 (5.8 to 11.4)
Antigua and Barbuda	0.4 (0.3 to 0.5)	0.3 (0.3 to 0.4)	8 (6.2 to 10.1)	7.1 (5.5 to 9)
Argentina	1.2 (1 to 1.5)	0.6 (0.5 to 0.8)	29.7 (24.8 to 34.8)	15.2 (12.3 to 18.1)
Armenia	2.2 (1.8 to 2.6)	2.3 (1.9 to 2.8)	51.1 (41.5 to 61.6)	50.9 (41.1 to 61.6)
Australia	1 (0.8 to 1.2)	0.4 (0.3 to 0.5)	22.1 (18.6 to 25.9)	7.7 (6.2 to 9.8)
Austria	1.3 (1.1 to 1.5)	0.8 (0.7 to 1)	29.5 (25.2 to 34.4)	18.2 (15.1 to 21.7)
Azerbaijan	0.7 (0.5 to 1)	0.7 (0.5 to 1)	18.3 (13.4 to 25.7)	16.1 (10.8 to 22.6)
Bahamas	0.2 (0.2 to 0.3)	0.2 (0.2 to 0.3)	5.7 (4.2 to 7.3)	5.4 (3.8 to 6.9)
Bahrain	2.5 (1.8 to 3.3)	1.4 (0.9 to 2)	51 (37.3 to 66.7)	27.4 (18 to 39.4)
Bangladesh	0.5 (0.3 to 0.8)	0.4 (0.2 to 0.7)	10.5 (6.3 to 16.7)	6.5 (3.7 to 13.3)
Barbados	0.4 (0.3 to 0.5)	0.3 (0.2 to 0.4)	7.2 (5.7 to 8.8)	5.6 (3.9 to 7.7)
Belarus	1.3 (1.1 to 1.5)	1 (0.8 to 1.2)	31.1 (26.9 to 35.4)	23.2 (18 to 28.4)
Belgium	2.3 (2 to 2.7)	1 (0.9 to 1.3)	49.8 (42.5 to 57.3)	22.5 (19 to 26.8)
Belize	0.3 (0.2 to 0.3)	0.3 (0.2 to 0.4)	5.8 (4.7 to 7)	6 (4.5 to 7.5)
Benin	0.2 (0.1 to 0.3)	0.1 (0.1 to 0.1)	4.6 (3.2 to 6.7)	2.3 (1.6 to 3.2)
Bermuda	1.2 (0.9 to 1.5)	0.8 (0.6 to 1)	25.2 (19.2 to 32.4)	16.3 (12.2 to 21.3)
Bhutan	0.2 (0.1 to 0.4)	0.2 (0.1 to 0.4)	4.2 (2.1 to 7.3)	3.4 (1.9 to 7.7)
Bolivia (Plurinational State of)	0.3 (0.2 to 0.5)	0.3 (0.2 to 0.4)	7 (4.7 to 10)	5.1 (3.3 to 7.4)
Bosnia and Herzegovina	1.1 (0.9 to 1.4)	1.5 (1.1 to 2.1)	25.9 (21.1 to 31.9)	33.6 (24.1 to 44.6)
Botswana	0.6 (0.4 to 0.8)	0.4 (0.3 to 0.6)	11.8 (8.2 to 15.9)	8.8 (6.3 to 12.4)
Brazil	1 (0.8 to 1.1)	0.5 (0.4 to 0.6)	20.1 (17.5 to 23)	10.4 (8.5 to 12.6)
Brunei Darussalam	1.1 (0.8 to 1.4)	0.5 (0.4 to 0.7)	22 (16.6 to 29.3)	10.6 (7.8 to 13.6)
Bulgaria	1.3 (1 to 1.5)	1.4 (1.1 to 1.8)	31.9 (26.9 to 37.7)	36.7 (28.8 to 46.7)
Burkina Faso	0.1 (0.1 to 0.2)	0.1 (0.1 to 0.1)	2.5 (1.7 to 3.5)	2 (1.4 to 2.8)
Burundi	0.4 (0.2 to 0.6)	0.2 (0.1 to 0.2)	8.2 (5.2 to 12.7)	3.6 (2.4 to 5.4)
Cabo Verde	0 (0 to 0.1)	0.2 (0.1 to 0.2)	0.6 (0.3 to 1.7)	3.6 (1.8 to 5.3)
Cambodia	0.6 (0.4 to 0.9)	0.5 (0.4 to 0.8)	12.4 (8.9 to 18.4)	11.1 (7.5 to 16.8)
Cameroon	0.2 (0.2 to 0.3)	0.2 (0.1 to 0.2)	5 (3.7 to 6.7)	3.8 (2.5 to 5.4)
Canada	1.8 (1.5 to 2.1)	0.8 (0.6 to 0.9)	39.7 (33.9 to 45.6)	14.8 (11.9 to 18.1)
Central African Republic	0.3 (0.2 to 0.5)	0.2 (0.2 to 0.3)	7.7 (4.9 to 11.5)	5.3 (3.6 to 7.6)

Chad	0.2 (0.1 to 0.3)	0.2 (0.1 to 0.3)	4.3 (2.7 to 6)	4 (2.4 to 5.9)
Chile	0.6 (0.4 to 0.7)	0.3 (0.2 to 0.4)	13.8 (11.2 to 16.7)	7.5 (6.1 to 9.1)
China	1.2 (0.8 to 1.5)	0.9 (0.7 to 1.2)	25.3 (16.8 to 31.7)	17.1 (12.7 to 23.4)
Colombia	0.4 (0.3 to 0.4)	0.1 (0.1 to 0.2)	7.7 (6.4 to 9.2)	2.9 (2.2 to 3.9)
Comoros	0.4 (0.2 to 0.6)	0.3 (0.1 to 0.5)	7.2 (4.4 to 11.4)	5.3 (2.8 to 9.1)
Congo	0.4 (0.3 to 0.6)	0.4 (0.3 to 0.6)	8.5 (5.2 to 12.4)	8.3 (5.7 to 12.1)
Cook Islands	0.6 (0.4 to 0.8)	0.5 (0.3 to 0.7)	14.5 (10.6 to 19)	11.6 (8.5 to 16.1)
Costa Rica	0.6 (0.5 to 0.7)	0.4 (0.3 to 0.5)	12.2 (10.3 to 14.4)	7.1 (5.6 to 8.9)
Coted'Ivoire	0.3 (0.2 to 0.4)	0.2 (0.1 to 0.3)	5.4 (3.6 to 7.3)	4.7 (3.1 to 6.8)
Croatia	2.1 (1.7 to 2.5)	1.8 (1.4 to 2.2)	42.6 (35.7 to 50.2)	38.2 (30.5 to 47.1)
Cuba	1.2 (1 to 1.4)	1 (0.8 to 1.3)	24.9 (21.3 to 28.9)	22.4 (17.8 to 27.2)
Cyprus	2.4 (1.7 to 3.3)	1.3 (0.9 to 1.8)	42.8 (32 to 57.3)	26.5 (19.2 to 36.2)
Czechia	1.8 (1.5 to 2.2)	1.3 (1 to 1.7)	43.3 (36.4 to 51.3)	29.9 (23.3 to 37.7)
Democratic People's Republic of Korea	0.6 (0.4 to 0.8)	0.5 (0.3 to 0.7)	13.6 (9.4 to 18.7)	11.9 (8.4 to 17.7)
Democratic Republic of the Congo	0.2 (0.1 to 0.3)	0.1 (0.1 to 0.2)	4.3 (2.9 to 6.2)	3.4 (2.3 to 5)
Denmark	2.4 (2 to 3.1)	1.5 (1.2 to 1.8)	52.2 (42.6 to 65)	29.6 (24.8 to 34.9)
Djibouti	0.5 (0.3 to 0.8)	0.5 (0.3 to 0.9)	10.3 (6.5 to 15.9)	10.1 (5.9 to 16.7)
Dominica	0.4 (0.3 to 0.6)	0.4 (0.3 to 0.5)	8.7 (6.4 to 11.8)	7.7 (5.4 to 11)
Dominican Republic	0.3 (0.2 to 0.4)	0.3 (0.2 to 0.4)	6.2 (4.7 to 8)	5.8 (3.9 to 8.4)
Ecuador	0.2 (0.2 to 0.3)	0.2 (0.1 to 0.2)	4.5 (3.6 to 5.4)	3.2 (2.3 to 4.3)
Egypt	2.6 (1.9 to 3.4)	1.6 (1 to 2.9)	63.7 (39.2 to 80.9)	34.2 (22.8 to 61)
El Salvador	0.1 (0.1 to 0.1)	0.1 (0.1 to 0.1)	1.8 (1.3 to 2.4)	2 (1.5 to 2.8)
Equatorial Guinea	0.3 (0.2 to 0.4)	0.3 (0.2 to 0.5)	6.3 (4 to 9.4)	6 (3.6 to 9.8)
Eritrea	0.2 (0.1 to 0.3)	0.1 (0.1 to 0.2)	4.3 (2.6 to 6.6)	3.3 (2.1 to 5)
Estonia	1 (0.9 to 1.2)	0.9 (0.7 to 1.1)	26.7 (22.6 to 31.3)	21 (17 to 25.4)
Eswatini	0.3 (0.2 to 0.4)	0.2 (0.1 to 0.3)	6.4 (4.5 to 8.8)	4.8 (3.1 to 7.2)
Ethiopia	0.1 (0.1 to 0.2)	0.1 (0.1 to 0.2)	3.3 (1.8 to 5.2)	2 (1.2 to 3.4)
Fiji	0.3 (0.2 to 0.4)	0.2 (0.1 to 0.3)	7.2 (4.9 to 9.4)	6.1 (3.4 to 8.8)
Finland	0.9 (0.7 to 1.1)	0.4 (0.3 to 0.5)	21.2 (17.7 to 24.9)	9 (7.2 to 11)
France	2 (1.7 to 2.3)	1.1 (0.9 to 1.3)	43.7 (37.7 to 50.2)	23.7 (19.2 to 28.3)
Gabon	0.3 (0.2 to 0.5)	0.3 (0.2 to 0.5)	7.2 (4.7 to 10.2)	7.1 (5 to 10.5)
Gambia	0.2 (0.1 to 0.3)	0.1 (0.1 to 0.2)	4.3 (2.8 to 5.9)	3.1 (2.1 to 4.5)
Georgia	1.2 (0.9 to 1.5)	2 (1.6 to 2.4)	31.3 (24.4 to 39.7)	46 (37.5 to 55.7)
Germany	1.7 (1.5 to 2)	0.9 (0.7 to 1.1)	37.6 (32.3 to 42.7)	19.2 (16 to 23.1)
Ghana	0.2 (0.1 to 0.3)	0.2 (0.1 to 0.3)	3.9 (2.7 to 5.4)	3.5 (2.3 to 5.2)
Greece	3.3 (2.8 to 3.7)	2.2 (1.9 to 2.6)	67.9 (59.7 to 76.1)	46.9 (40.3 to 54.6)
Greenland	2 (1.3 to 2.5)	1.1 (0.7 to 1.5)	44.7 (29.2 to 57.4)	23.6 (16 to 32.8)
Grenada	0.3 (0.2 to 0.4)	0.3 (0.2 to 0.4)	7.6 (5.7 to 9.6)	6.7 (5 to 8.5)
Guam	0.2 (0.2 to 0.3)	0.2 (0.2 to 0.3)	6.4 (4.7 to 8.5)	6.6 (4.9 to 8.4)
Guatemala	0.1 (0.1 to 0.2)	0.1 (0.1 to 0.1)	2.5 (2 to 3.2)	1.5 (1.1 to 1.9)
Guinea	0.3 (0.2 to 0.5)	0.4 (0.3 to 0.6)	7.1 (4.8 to 10)	8.1 (5.3 to 12)
Guinea-Bissau	0.2 (0.1 to 0.2)	0.2 (0.1 to 0.2)	3.6 (2 to 5.5)	3.8 (2.5 to 5.7)

Guyana	0.3 (0.2 to 0.3)	0.2 (0.1 to 0.3)	5.8 (4.5 to 7.3)	4.8 (3.3 to 6.7)
Haiti	0.3 (0.2 to 0.4)	0.2 (0.1 to 0.3)	6.2 (3.9 to 9.4)	4.1 (2.5 to 6)
Honduras	0.1 (0.1 to 0.1)	0.1 (0.1 to 0.2)	2.2 (1.5 to 3)	2.7 (1.7 to 4.2)
Hungary	1.7 (1.4 to 2)	1.4 (1.1 to 1.8)	42.1 (35.5 to 48.9)	36.4 (29.3 to 44.7)
Iceland	1.7 (1.4 to 1.9)	0.7 (0.6 to 0.9)	35.9 (30.3 to 41.6)	15.1 (12 to 19.2)
India	0.3 (0.2 to 0.4)	0.2 (0.2 to 0.3)	6.5 (4.4 to 8.5)	4.7 (3.7 to 6)
Indonesia	0.3 (0.2 to 0.4)	0.3 (0.2 to 0.6)	6.4 (4.9 to 8.6)	7.1 (4.4 to 12.6)
Iran (Islamic Republic of)	0.5 (0.4 to 0.7)	0.5 (0.4 to 0.6)	11.6 (8.3 to 15.3)	10.8 (8 to 13.4)
Iraq	1.7 (1.2 to 2.4)	1.8 (1.2 to 2.6)	36.7 (25 to 51.5)	37.4 (24.6 to 54.5)
Ireland	1.8 (1.5 to 2.1)	0.7 (0.6 to 0.9)	35.8 (30.8 to 41)	13.9 (11 to 17)
Israel	1.5 (1.2 to 1.8)	0.9 (0.7 to 1.1)	31.2 (26.5 to 36.7)	17.7 (14.2 to 21.6)
Italy	2.6 (2.2 to 2.9)	1.2 (1 to 1.4)	58.5 (50.2 to 66.1)	25.6 (21.2 to 30.1)
Jamaica	0.5 (0.4 to 0.6)	0.4 (0.3 to 0.6)	9.8 (8.1 to 12)	8.7 (6.1 to 12.1)
Japan	0.9 (0.7 to 1)	0.5 (0.4 to 0.6)	17.4 (15.4 to 19.5)	10.6 (8.9 to 12.5)
Jordan	1.4 (1 to 1.8)	1 (0.7 to 1.4)	29 (21.2 to 38.4)	21.4 (15 to 30.2)
Kazakhstan	0.7 (0.5 to 1)	0.5 (0.4 to 0.6)	18.7 (13.5 to 25.9)	12.5 (9.8 to 15.5)
Kenya	0.1 (0.1 to 0.2)	0.1 (0.1 to 0.1)	2.4 (1.5 to 3.6)	2.3 (1.7 to 3)
Kiribati	0.1 (0.1 to 0.2)	0.2 (0.1 to 0.2)	3.3 (2.6 to 4.1)	3.7 (2.7 to 4.7)
Kuwait	0.7 (0.6 to 0.9)	0.8 (0.6 to 1)	15.8 (12.5 to 19.3)	16.7 (12.4 to 21.5)
Kyrgyzstan	0.6 (0.5 to 0.7)	0.5 (0.4 to 0.7)	14.9 (11.5 to 18)	12.6 (9.8 to 16.1)
Lao People's Democratic Republic	0.6 (0.3 to 0.9)	0.5 (0.3 to 0.7)	12.3 (7.4 to 19.3)	9.4 (6 to 14.4)
Latvia	1.2 (1 to 1.4)	1.3 (1.1 to 1.6)	29.7 (25.7 to 34)	31.5 (25.3 to 38.6)
Lebanon	3.4 (2.2 to 4.8)	2.9 (2.1 to 4)	71.8 (45.7 to 101)	58.9 (43.1 to 78.6)
Lesotho	0.4 (0.3 to 0.6)	0.6 (0.3 to 0.8)	7.9 (5.5 to 11.6)	12.7 (7.4 to 19.2)
Liberia	0.2 (0.1 to 0.3)	0.1 (0.1 to 0.2)	3.8 (2.5 to 5.5)	2.6 (1.7 to 3.9)
Libya	1.8 (1.1 to 2.6)	2 (1.4 to 2.9)	36.5 (24.3 to 53.2)	41 (28.3 to 58.8)
Lithuania	1.4 (1.1 to 1.6)	1.1 (0.9 to 1.4)	30.4 (25.3 to 35)	24.8 (20.3 to 29.6)
Luxembourg	1.8 (1.5 to 2.2)	0.9 (0.7 to 1.2)	41.2 (34 to 48.6)	19.7 (15.5 to 24.3)
Madagascar	0.3 (0.2 to 0.4)	0.1 (0.1 to 0.2)	5.1 (3.6 to 6.9)	2.3 (1.5 to 3.4)
Malawi	1.1 (0.8 to 1.5)	1.2 (0.9 to 1.7)	21.2 (15.9 to 27.3)	24.5 (16.7 to 33.6)
Malaysia	0.8 (0.5 to 1.2)	0.7 (0.5 to 1)	16.7 (9.7 to 23.4)	13.7 (9.2 to 18.5)
Maldives	0.8 (0.5 to 1.1)	0.5 (0.3 to 0.6)	14.1 (9.2 to 20.6)	7.7 (5.7 to 10.2)
Mali	0.8 (0.5 to 1)	1 (0.7 to 1.5)	14.2 (10.2 to 18.8)	19 (13.1 to 27.1)
Malta	1.9 (1.5 to 2.2)	0.8 (0.6 to 1)	41.6 (35 to 48.9)	18.8 (15.3 to 23.4)
Marshall Islands	0.3 (0.2 to 0.5)	0.4 (0.2 to 0.5)	7.4 (4.8 to 11.1)	8.8 (5.6 to 12.8)
Mauritania	0.2 (0.2 to 0.3)	0.2 (0.1 to 0.3)	5.2 (3.6 to 7.5)	3.4 (1.9 to 5.5)
Mauritius	1.2 (1 to 1.4)	0.6 (0.5 to 0.7)	26 (22.3 to 29.8)	12 (10.3 to 13.9)
Mexico	0.4 (0.4 to 0.5)	0.2 (0.1 to 0.2)	8.2 (6.9 to 9.6)	3.7 (3 to 4.6)
Micronesia (Federated States of)	0.4 (0.3 to 0.6)	0.4 (0.3 to 0.6)	10.8 (7.3 to 15.2)	11.4 (7.4 to 15.9)
Monaco	1.7 (1 to 2.4)	1.4 (0.6 to 3.4)	36.5 (21.8 to 54.3)	29.1 (13 to 73.5)
Mongolia	0.4 (0.3 to 0.6)	0.3 (0.2 to 0.4)	10.3 (6.4 to 14.9)	7.3 (5.2 to 10.5)
Montenegro	1.4 (1 to 1.8)	1.5 (1.1 to 2)	32.4 (24.1 to 42.3)	33.9 (25 to 45.6)

Morocco	0.3 (0.2 to 0.4)	0.3 (0.2 to 0.4)	6.7 (4.6 to 9)	5.6 (3.8 to 8)
Mozambique	0.3 (0.2 to 0.4)	0.3 (0.2 to 0.4)	5.5 (3.7 to 7.9)	5.6 (4 to 7.9)
Myanmar	0.6 (0.4 to 0.8)	0.3 (0.2 to 0.5)	12.2 (9 to 16.8)	5.7 (3.9 to 9.2)
Namibia	0.3 (0.2 to 0.4)	0.3 (0.2 to 0.4)	5.8 (4.3 to 7.5)	5 (3.8 to 6.6)
Nauru	0.6 (0.4 to 0.8)	0.5 (0.3 to 0.7)	13.5 (8.2 to 19.4)	12.3 (7.2 to 18.1)
Nepal	0.4 (0.3 to 0.7)	0.3 (0.2 to 0.6)	7.9 (4.9 to 12.5)	5.2 (3.3 to 10.3)
Netherlands	2.4 (2 to 2.7)	1.2 (1 to 1.4)	49.9 (43.1 to 56.2)	22.1 (18.5 to 26.8)
New Zealand	1.1 (0.9 to 1.4)	0.6 (0.5 to 0.8)	24.4 (20.4 to 28.6)	12.3 (9.8 to 15.2)
Nicaragua	0.1 (0.1 to 0.1)	0.1 (0.1 to 0.1)	2 (1.5 to 2.6)	1.8 (1.3 to 2.4)
Niger	0.1 (0.1 to 0.2)	0.1 (0 to 0.1)	1.9 (1.2 to 3.1)	1.4 (0.8 to 2.5)
Nigeria	0 (0 to 0.1)	0 (0 to 0)	0.7 (0.5 to 1)	0.5 (0.3 to 0.7)
Niue	0.3 (0.2 to 0.4)	0.3 (0.2 to 0.5)	7.6 (5 to 10.2)	8.3 (5.4 to 11.7)
North Macedonia	1.8 (1.4 to 2.3)	1.8 (1.3 to 2.3)	40.3 (31.7 to 50.4)	37.9 (27.5 to 49.8)
Northern Mariana Islands	0.4 (0.2 to 0.6)	0.5 (0.4 to 0.6)	8.5 (5.9 to 14.1)	11.9 (9.1 to 15.3)
Norway	1.8 (1.6 to 2.2)	0.7 (0.5 to 0.8)	38.8 (34.1 to 44.3)	13 (10.5 to 15.7)
Oman	0.3 (0.2 to 0.4)	0.2 (0.1 to 0.3)	7 (4.5 to 10.2)	4.6 (3.1 to 6.2)
Pakistan	1.6 (1.1 to 2.1)	1.3 (0.9 to 1.8)	30.7 (22.9 to 39.9)	24.9 (17.6 to 36.4)
Palau	0.2 (0.1 to 0.2)	0.1 (0.1 to 0.2)	3.6 (2.6 to 5)	3 (2.1 to 4.1)
Palestine	1.5 (1 to 2)	1.1 (0.8 to 1.5)	30 (20.4 to 42.3)	23.8 (17.7 to 31.8)
Panama	0.2 (0.2 to 0.3)	0.1 (0.1 to 0.2)	4.3 (3.6 to 5.2)	2.9 (2.1 to 3.8)
Papua New Guinea	0.2 (0.1 to 0.3)	0.2 (0.1 to 0.3)	4.4 (2.3 to 6.8)	4.2 (2.3 to 6.6)
Paraguay	0.5 (0.4 to 0.6)	0.4 (0.3 to 0.6)	8.7 (6.6 to 11.3)	8.2 (5.6 to 11.4)
Peru	0.2 (0.1 to 0.2)	0.1 (0.1 to 0.2)	3 (2.1 to 4.1)	2.4 (1.5 to 3.6)
Philippines	0.3 (0.2 to 0.4)	0.3 (0.2 to 0.4)	6.8 (5.2 to 8.7)	5.8 (4.4 to 8.2)
Poland	2 (1.7 to 2.2)	1.9 (1.5 to 2.2)	48.7 (42.9 to 54.1)	41.9 (34.6 to 49.8)
Portugal	1.2 (1 to 1.4)	0.7 (0.6 to 0.9)	26.4 (22.2 to 30.8)	18 (14.6 to 21.7)
Puerto Rico	0.5 (0.4 to 0.7)	0.4 (0.3 to 0.5)	10.7 (8.2 to 13.6)	7.8 (5.7 to 10.4)
Qatar	1.1 (0.8 to 1.6)	0.5 (0.3 to 0.9)	24.7 (17.4 to 34.7)	12.1 (7.5 to 18.8)
Republic of Korea	1.2 (0.9 to 1.5)	0.7 (0.5 to 0.9)	24.7 (18.8 to 30.4)	12.3 (8.8 to 15.9)
Republic of Moldova	1 (0.8 to 1.1)	1 (0.8 to 1.2)	23.8 (19.2 to 27.9)	25.6 (21.3 to 29.8)
Romania	1.2 (1 to 1.4)	1.3 (1 to 1.6)	29.6 (25.1 to 34.1)	32.1 (26.1 to 38.7)
Russian Federation	1.1 (0.9 to 1.2)	0.9 (0.7 to 1)	27.4 (24.3 to 30.6)	21.9 (18.2 to 25.6)
Rwanda	0.7 (0.5 to 1)	0.6 (0.4 to 0.9)	13.6 (9.7 to 18.9)	10.6 (7 to 16.5)
Saint Kitts and Nevis	0.4 (0.3 to 0.5)	0.3 (0.2 to 0.4)	8.1 (6.3 to 10.3)	6 (4.3 to 8.2)
Saint Lucia	0.6 (0.5 to 0.8)	0.4 (0.3 to 0.5)	13 (10.4 to 16.1)	8.2 (6.1 to 10.9)
Saint Vincent and the Grenadines	0.3 (0.3 to 0.4)	0.3 (0.2 to 0.4)	7 (5.6 to 8.7)	6.9 (5.4 to 8.8)
Samoa	0.4 (0.3 to 0.6)	0.4 (0.3 to 0.5)	10.1 (7.4 to 14.5)	8.4 (5.7 to 12)
San Marino	2.4 (1.7 to 3.1)	0.9 (0.6 to 1.4)	49.4 (36.6 to 65.5)	19.4 (11.5 to 30.1)
Sao Tome and Principe	0.2 (0.1 to 0.3)	0.3 (0.2 to 0.4)	4.4 (3.1 to 6)	6.2 (3.9 to 9.4)
Saudi Arabia	0.3 (0.2 to 0.4)	0.3 (0.2 to 0.5)	6.8 (4.2 to 10.6)	6.8 (4.2 to 12.6)
Senegal	0.2 (0.2 to 0.3)	0.2 (0.1 to 0.2)	6 (4.1 to 8.3)	3.8 (2.5 to 5.7)
Serbia	1.7 (1.3 to 2.2)	1.4 (1 to 1.9)	36.6 (27.7 to 47.8)	33.8 (24.4 to 45.3)
Seychelles	1.5 (1.1 to 1.9)	1.1 (0.8 to 1.4)	31.7 (24.5 to 40.3)	21.5 (16.8 to 28.1)

Sierra Leone	0.3 (0.2 to 0.4)	0.2 (0.1 to 0.2)	5.7 (4 to 7.7)	3.5 (2.5 to 5.1)
Singapore	0.5 (0.4 to 0.6)	0.2 (0.2 to 0.3)	11.5 (9.4 to 13.8)	4.2 (3.3 to 5.2)
Slovakia	1.5 (1.2 to 2)	1.1 (0.8 to 1.5)	36.3 (28.6 to 47)	24.8 (18.1 to 33.5)
Slovenia	1 (0.9 to 1.2)	0.9 (0.7 to 1.2)	26.6 (22.1 to 31.2)	21.6 (16.8 to 27.1)
Solomon Islands	0.4 (0.2 to 0.6)	0.4 (0.2 to 0.6)	9.8 (4.7 to 15.1)	10.5 (5.9 to 16)
Somalia	0.3 (0.2 to 0.5)	0.2 (0.1 to 0.3)	5.7 (3.4 to 9.8)	4.2 (2.3 to 7)
South Africa	0.6 (0.4 to 0.8)	0.3 (0.3 to 0.4)	12.1 (8.8 to 16.7)	7.9 (6.3 to 9.7)
South Sudan	0.3 (0.2 to 0.4)	0.2 (0.1 to 0.4)	6.3 (4.1 to 9.5)	4.8 (3 to 7.4)
Spain	2.7 (2.4 to 3.1)	1.4 (1.1 to 1.7)	61.7 (53 to 69.8)	30.2 (24.7 to 36.3)
Sri Lanka	0.3 (0.2 to 0.4)	0.2 (0.1 to 0.2)	5.8 (4.4 to 7.8)	3.3 (1.8 to 4.8)
Sudan	0.7 (0.4 to 1.2)	0.5 (0.4 to 0.8)	15.2 (8.5 to 26.7)	11.8 (7.9 to 17.1)
Suriname	0.4 (0.3 to 0.5)	0.3 (0.2 to 0.4)	9.2 (6.9 to 12.1)	7.3 (4.8 to 10.3)
Sweden	1.2 (1 to 1.4)	0.8 (0.6 to 1)	24.9 (21.1 to 28.9)	15.2 (12.1 to 18.7)
Switzerland	0.9 (0.8 to 1.1)	0.9 (0.7 to 1.1)	20.2 (16.9 to 23.5)	17.2 (13.9 to 20.8)
Syrian Arab Republic	0.9 (0.6 to 1.2)	0.8 (0.5 to 1.1)	18.7 (13.4 to 25.4)	15.9 (10.5 to 22.8)
Taiwan (Province of China)	1.3 (1.1 to 1.5)	0.9 (0.7 to 1)	27.7 (23.6 to 31.5)	17.3 (14.4 to 20.1)
Tajikistan	0.3 (0.2 to 0.5)	0.2 (0.1 to 0.2)	7.2 (4.7 to 11)	3.7 (2.3 to 5.5)
Thailand	1 (0.7 to 1.3)	0.6 (0.4 to 0.8)	20.5 (14.5 to 26.2)	11.9 (8.4 to 16.7)
Timor-Leste	0.3 (0.2 to 0.4)	0.3 (0.2 to 0.4)	5.7 (3.4 to 9.1)	5.2 (3.4 to 8.2)
Togo	0.5 (0.3 to 0.6)	0.3 (0.2 to 0.4)	9.4 (6.7 to 12.7)	6.2 (4.3 to 8.9)
Tokelau	0.4 (0.2 to 0.5)	0.3 (0.2 to 0.4)	8.2 (5.2 to 12)	7.2 (4.5 to 10.3)
Tonga	0.4 (0.3 to 0.7)	0.4 (0.3 to 0.7)	9.5 (5.8 to 15.5)	9.6 (5.7 to 15.5)
Trinidad and Tobago	0.4 (0.4 to 0.5)	0.3 (0.2 to 0.4)	9.3 (7.7 to 10.9)	6.7 (4.7 to 8.9)
Tunisia	1.5 (1.1 to 2.1)	1.3 (0.8 to 1.9)	29.7 (20.9 to 41)	27 (16.9 to 39.5)
Turkey	1.9 (1.4 to 2.7)	1.3 (0.9 to 1.8)	44.7 (31.8 to 61.1)	29.5 (21.2 to 40)
Turkmenistan	0.4 (0.3 to 0.5)	0.3 (0.2 to 0.4)	10.6 (8.9 to 12.6)	6.9 (4.9 to 9.4)
Tuvalu	0.3 (0.2 to 0.5)	0.4 (0.2 to 0.5)	8.6 (5.8 to 11.8)	9 (6 to 12.4)
Uganda	0.2 (0.2 to 0.3)	0.2 (0.1 to 0.3)	4.8 (3.4 to 6.6)	4 (2.9 to 5.6)
Ukraine	1 (0.8 to 1.4)	0.9 (0.6 to 1.2)	25.1 (19.3 to 33.7)	21.8 (14.1 to 30.9)
United Arab Emirates	1.2 (0.6 to 2.3)	0.8 (0.6 to 1.2)	27.1 (14.1 to 52.7)	16.1 (11.2 to 22.7)
United Kingdom	2.6 (2.2 to 2.9)	1.1 (0.9 to 1.4)	54.7 (47.6 to 61.8)	21.3 (17.6 to 25.3)
United Republic of Tanzania	0.4 (0.3 to 0.6)	0.3 (0.2 to 0.4)	8.5 (6.1 to 12.2)	5.2 (3.5 to 7.6)
United States of America	1.1 (0.9 to 1.3)	0.8 (0.6 to 1)	26 (22.3 to 29.8)	18.1 (14.9 to 21.8)
United States Virgin Islands	0.3 (0.2 to 0.4)	0.2 (0.1 to 0.2)	6 (4.2 to 8.5)	3.4 (2.2 to 5)
Uruguay	1.6 (1.4 to 1.9)	1.3 (1 to 1.5)	38.9 (32.9 to 45.1)	28.4 (23.2 to 33.8)
Uzbekistan	0.2 (0.1 to 0.2)	0.2 (0.2 to 0.3)	4.1 (2.8 to 5.6)	5.9 (4.2 to 7.8)
Vanuatu	0.3 (0.2 to 0.4)	0.2 (0.1 to 0.3)	6.7 (3.9 to 10.1)	5.4 (3.3 to 8.3)
Venezuela (Bolivarian Republic of)	0.4 (0.3 to 0.5)	0.3 (0.2 to 0.4)	8.4 (6.9 to 9.9)	5.4 (3.8 to 7.4)
Viet Nam	0.4 (0.3 to 0.5)	0.4 (0.3 to 0.5)	7.7 (5.3 to 10.3)	8.4 (6.3 to 10.8)
Yemen	0.8 (0.5 to 1.3)	0.8 (0.6 to 1.2)	19.5 (11.2 to 30.8)	18.7 (12.5 to 25.9)
Zambia	0.3 (0.3 to 0.4)	0.3 (0.2 to 0.5)	6.7 (5.1 to 9)	6.6 (3.8 to 12.2)

Zimbabwe	1.5 (1.1 to 2)	1.5 (1.1 to 2)	30.8 (22.8 to 40.5)	32.1 (23.8 to 43.2)

ASMR：Age-standardized mortality rate; ASDR: Age-standardized DALYs rate

2. **Supplementary figure 1.** The temporal change of the mortality rate in bladder cancer attributable to smoking across age groups and sexes.

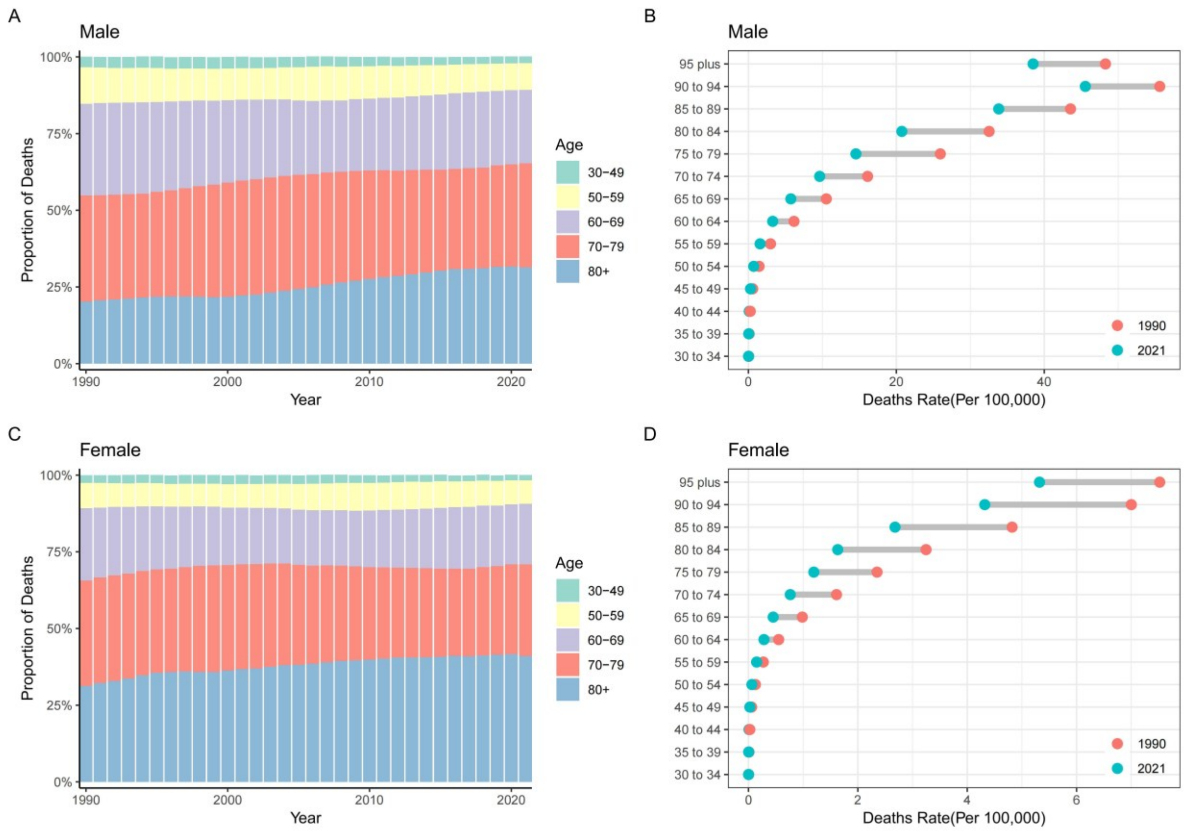

=

3. **Supplementary figure 2.** The temporal change of the DALYs in bladder cancer attributable to smoking across age groups and sexes.

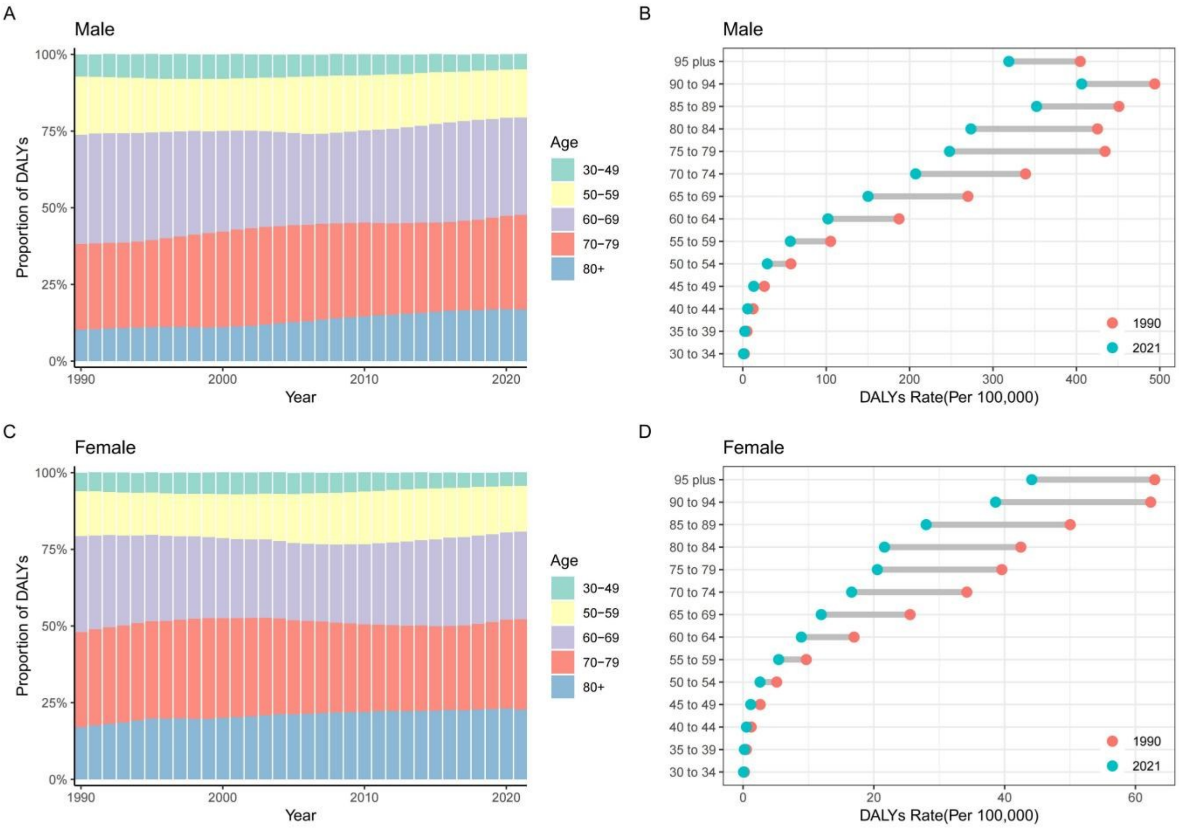

4. **Supplementary figure 3. The relationship between SDI levels and (A)the age-standardized mortality rate and (B)the estimated annual percentage change of ASMR for bladder cancer attributable to smoking from 1990 to 2021.**

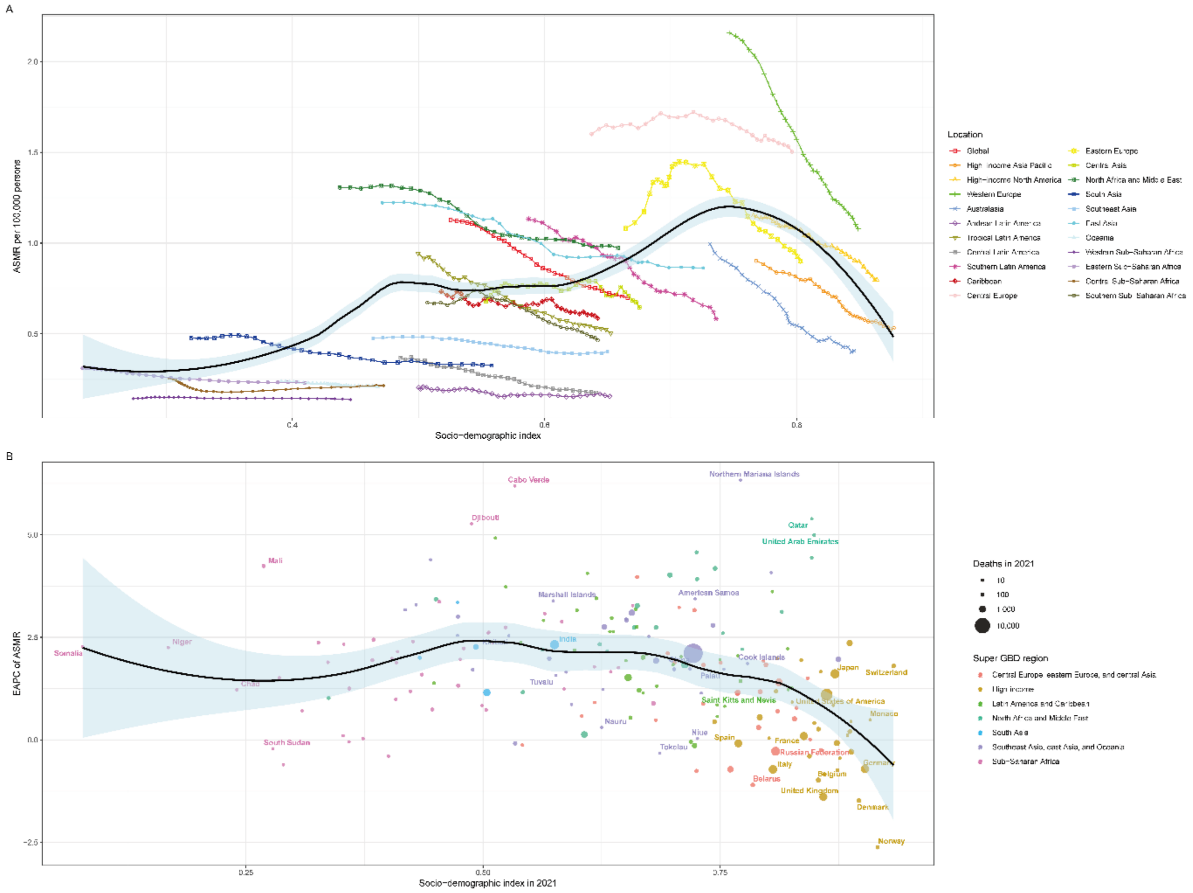

5. **Supplementary figure 4. The relationship between SDI levels and (A)the age-standardized DALYs rate and (B)the estimated annual percentage change of ASDR for bladder cancer attributable to smoking from 1990 to 2021.**

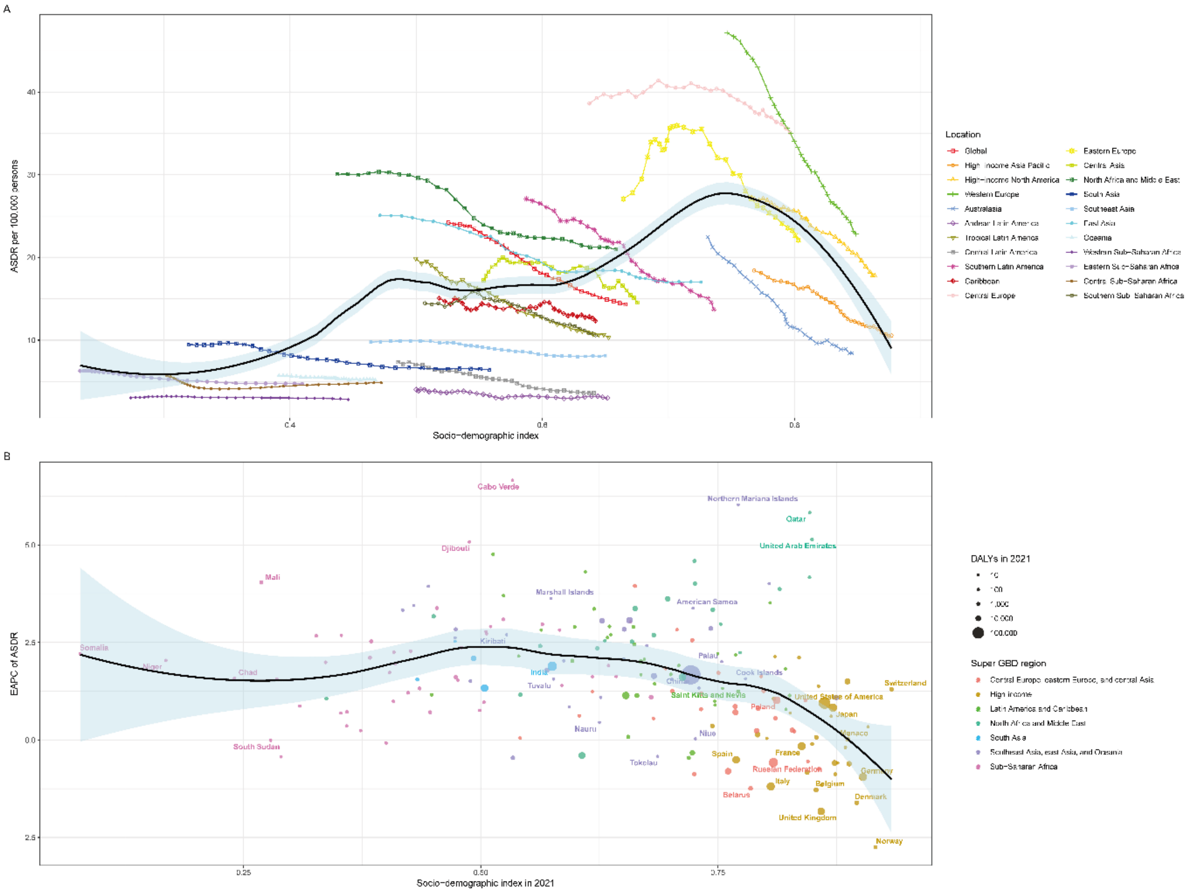

6. **Supplementary figure 5.** The temporal change of the mortality rate in bladder cancer attributable to smoking across age groups and SDI.

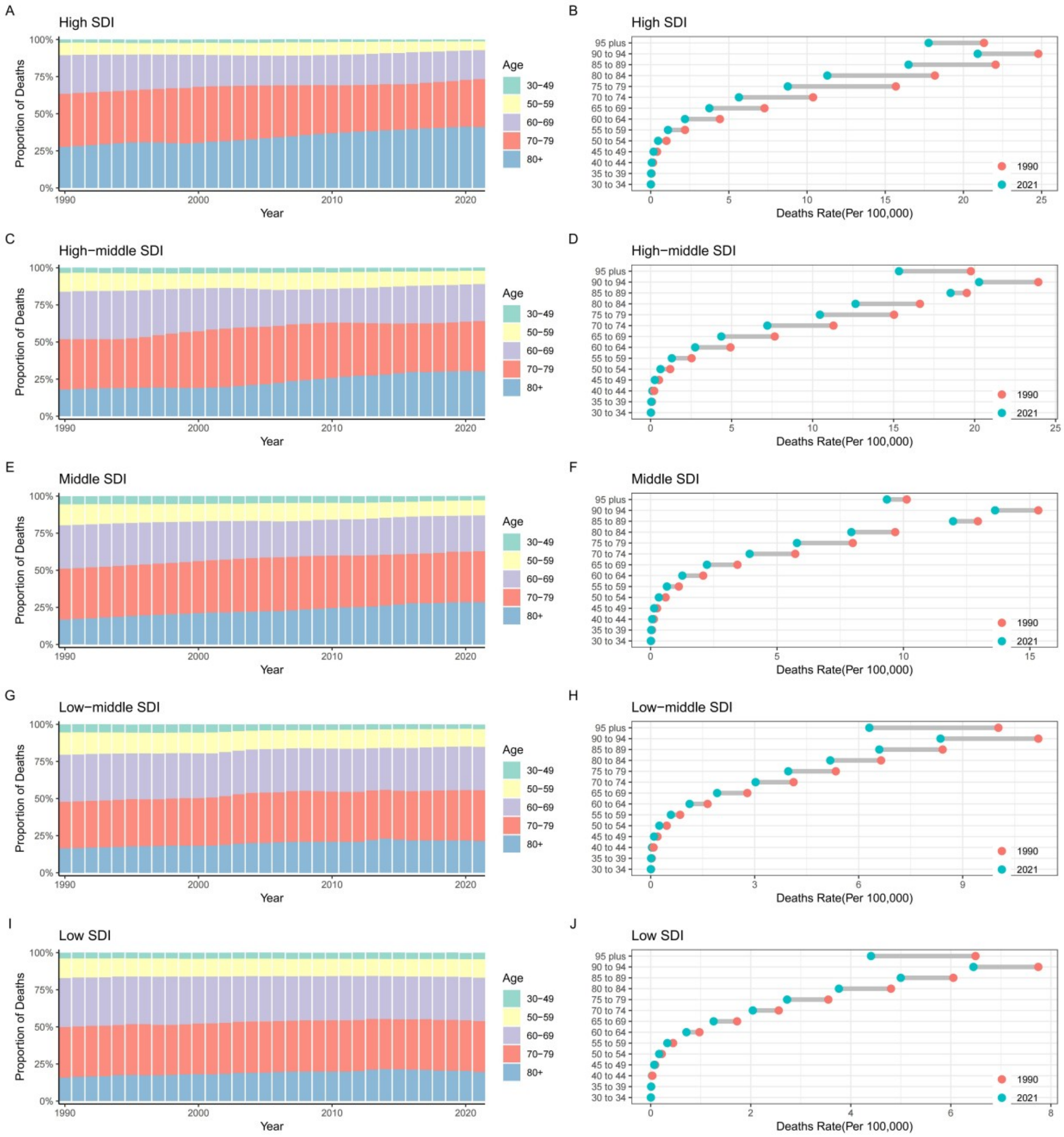

7. **Supplementary figure 6.** The temporal change of the DALYs in bladder cancer attributable to smoking across age groups and SDI.

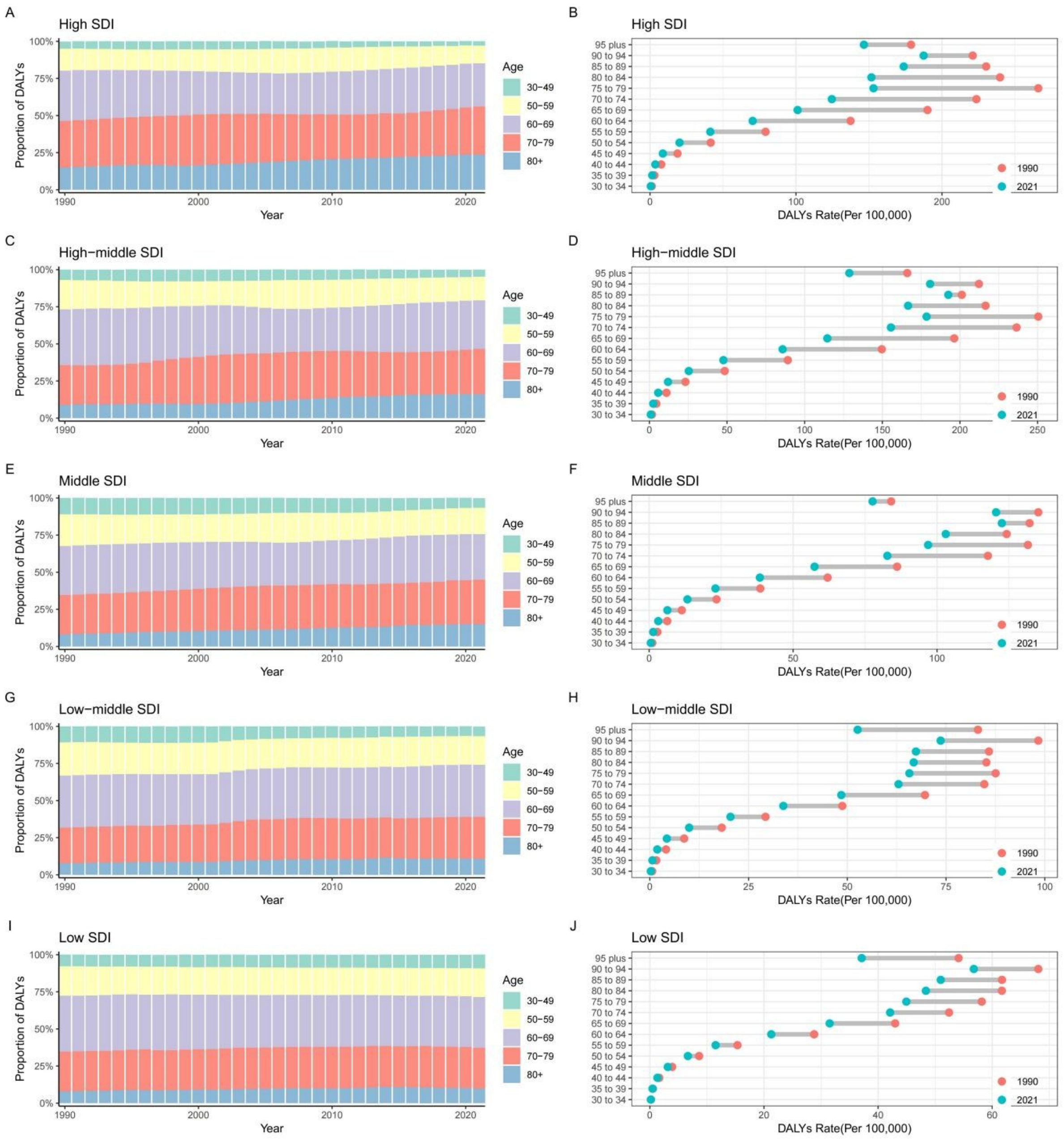

8. **Supplementary figure 7. Maps showing age-standardized changes in mortality rates (A, B) and DALYs (C, D) among people with bladder cancer attributable to smoking from 1990 to 2021.**

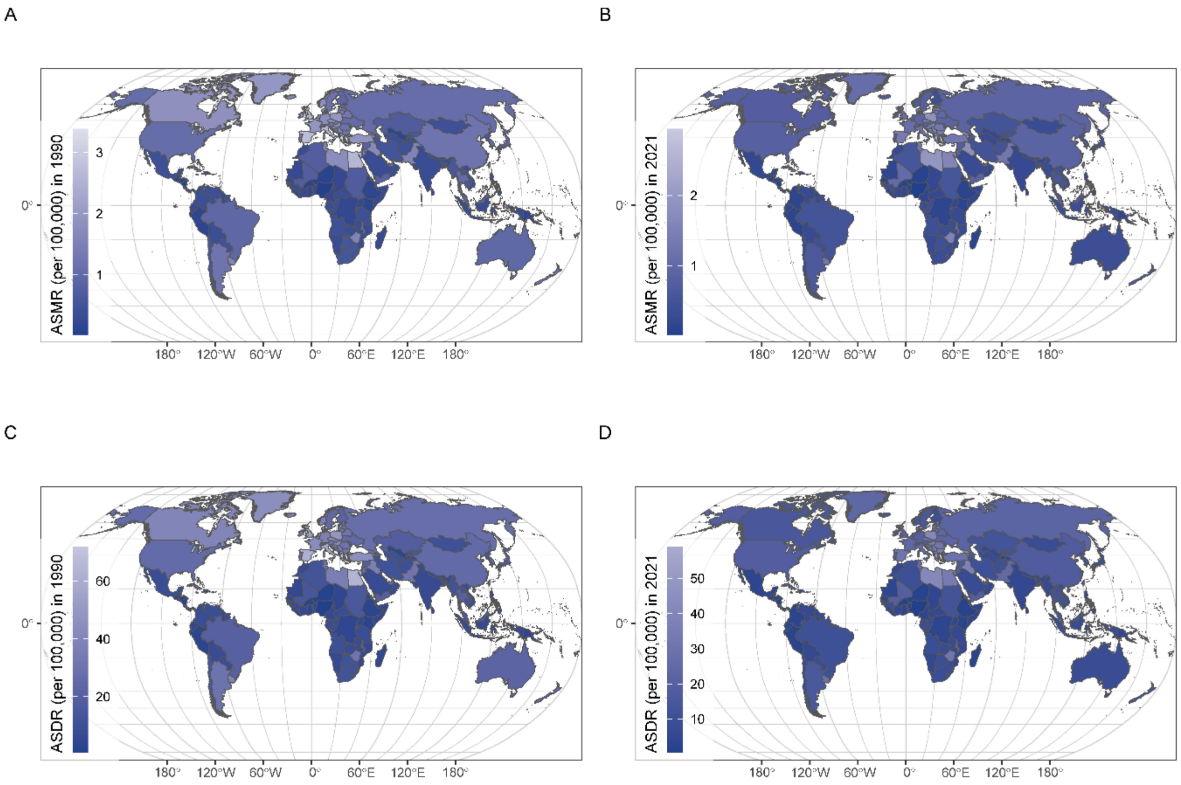

© 2025 Yu Q. et al.
